# Spontaneous Growth Induced by a Biologically Oriented V Class Restoration (BOVR) Technique: A 3D Prospective Study

**DOI:** 10.3390/dj13070296

**Published:** 2025-06-30

**Authors:** Davide Farronato, Giuseppe Messina, Luciano Laveglia, Pietro Mario Pasini, Lorenzo Azzi, Marco Farronato

**Affiliations:** 1 Department of Medicine and Technological Innovation, School of Dentistry, University of Insubria, 21100 Varese, Italy; 2 Department of Medicine and Surgery, School of Dentistry, University of Insubria, 21100 Varese, Italy; 3 Independent Researcher, 21100 Varese, Italy; 4 Azienda Socio-Sanitaria Territoriale (ASST) dei Sette Laghi, 21100 Varese, Italy; 5 Department of Biomedical, Surgical and Dental Sciences, School of Dentistry, University of Milan, 20100 Milan, Italy; 6 Fondazione IRCCS Cà Granda, Ospedale Maggiore Policlinico, 20122 Milan, Italy

**Keywords:** gingival recession, conservative treatment, oral hygiene, cemento-enamel junction, tooth abrasion

## Abstract

**Background:** The behavior of soft tissues following recession type 1 (RT1) and/or non-carious cervical lesions (NCCLs) treated with class V restorations is not well understood. These conditions cause both functional and esthetic issues. Recent studies show that increased cervical thickness can influence gingival tissue response. This suggests that restoration design has a key impact. This study aims to evaluate the effect of tooth shape modification on gingival tissue response and periodontal health with 3D analysis. **Methods**: Seven patients with buccal gingival recession and NCCL were selected, resulting in 50 treated teeth. Patients underwent class V buccal restorations using the BOVR technique. Three-dimensional evaluation through scanned dental impressions was performed at baseline and at T1 to monitor tissue profile changes in the buccal zenith sagittal plane. The average observation period was 4 months. Following the assessment, linear measurements were calculated according to standard planes. These measurements aimed to monitor transverse and axial tissue modifications. Probing depth, plaque index, and bleeding index were also recorded. **Results**: Increased tooth thickness led to tissue alteration. Greater composite thickness was significantly associated with an increase in tissue thickness (*p* ≤ 0.001) and gingival creeping (*p* ≤ 0.001) at the free gingival margin. Periodontal health remained unaffected, and 50% of the teeth required no additional surgical treatment due to satisfactory outcomes. **Conclusions**: Class V restorations that increase cervical thickness may promote soft tissue volume gain over a 4-month period without compromising periodontal health. A 4-month observation period is recommended before considering the surgical correction.

## 1. Introduction

Gingival recession is a common clinical condition, with global prevalence estimates ranging from 40% to 100% and increasing with age and the presence of periodontal disease [1,2]. A recent study in the Italian adult population reported that 57.2% had mid-buccal recessions of ≥1 mm, most frequently affecting premolars and canines [2].

This condition may result from traumatic factors such as improper brushing techniques or from periodontal inflammation caused by plaque accumulation [2,3]. An epidemiological study reported that traumatic brushing accounted for 43% of cases, while plaque-induced factors were responsible for 44% [3]. Current treatment typically involves periodontal plastic surgery. Cairo defined three gingival recession types (RT1, RT2, and RT3), each requiring different periodontal surgical approaches. These techniques aim to prevent and treat anatomical, infectious, or developmental issues that can damage the gum, alveolar mucosa, or bone [4]. Gingival recessions often co-occur alongside non-carious cervical lesions (NCCLs) [5]. NCCLs involve the loss of hard dental tissue, affecting enamel, dentin, and/or cementum. Unlike caries, recessions lack an infectious component [6,7,8]. Although no consensus exists regarding treatment, one common approach involves the use of conservative restorations, without additional intervention. NCCLs are defined as the loss of dental hard tissue at the cementoenamel junction (CEJ). These lesions are not caused by bacterial activity, but are linked to multiple factors, including abrasion, erosion, and abfraction. In the presence of gingival recession, NCCLs can worsen both esthetic and functional problems [6]. It corrects the NCCL and camouflages the gingival recession by modifying the cervical contour. Another approach is periodontal, which surgically corrects the gingival recession and conceals the cervical lesion [9]. Recently, a combined conservative periodontal approach gained widespread clinical acceptance [9,10,11,12]. The literature outlines different protocols for this combined approach: some authors restore the original shape altered by the NCCL [11], while others extend the restoration 1 mm apically beyond the CEJ [13]. In other cases, the restoration is extended 1 mm beyond the maximum root coverage (MRC) [14]. The goal remains to restore the tooth’s original shape.

A classification system divides NCCLs with gingival recessions into five classes. It enumerates the most effective treatments: exclusively conservative, conservative and surgical, or surgical only [9]. While composite restorations typically extend to the MRC, there is no consensus on the ideal timing when conservative and surgical treatments are performed separately. Most described procedures align the timing of restoration with surgery, yet no studies specifically discuss this timing.

A recent approach proposes the treatment of NCCLs not only for structural restoration, but also to promote a favorable gingival tissue response in thickness and position. The coronally dynamic technique is a minimally invasive restorative protocol developed specifically for NCCLs associated with gingival recession. This technique includes a slightly over-contoured cervical restoration that replicates the natural convexity of the CEJ. It is combined with gentle sulcular de-epithelialization, also known as “gingitage”. It aims to stabilize a blood clot within the gingival sulcus and encourage coronal migration of the gingival margin [15]. This minimally invasive technique applies biologically oriented preparation technique (BOPT) principles to modify gum contours [16]. Monitoring is typically performed 15 days after reconstruction. The results suggest a link between dental shape and tissue maturation volume. Even with minimally invasive techniques, this effect appears relevant. However, tissue changes may require a longer period to fully reflect the influence of the modified tooth shape on gingival volume.

In class V restorations for NCCL repair, the cervical emergence profile (dentine-composite junction, DTC) affects hygiene maintenance. Increased contours may lead to plaque retention and biological complications [17]. Some studies suggest a correlation between higher plaque index and increased contour [18,19].

This study aimed to evaluate whether these hygiene-related findings apply to natural teeth with modified thickness.

The biologically oriented V class restoration (BOVR) technique is proposed for cases of flat or coronal CEJ migration caused by erosion, abrasion, abfraction, or genetic factors [20]. It involves a composite restoration that does not recreate the original tooth shape. Instead, it forms a new cervical shape with transverse thickness matching the gingival thickness. This approach differs from traditional methods [21]. This approach aims to evaluate whether the new cervical morphology induces gingival tissue changes over a four-month period.

The null hypothesis tests that no correlation exists between tissue adaptation and tooth shape and whether there is a correlation between tissue adaptation and tooth shape. It measures both in sagittal and horizontal planes at the buccal zenith after BOVR restoration [19]. The observation period was extended to allow tissue adaptation and was set at 4 months (±1). The secondary aim was to determine whether increased tooth thickness in class V restorations affects periodontal inflammation indicators. These include bleeding on probing (BOP), plaque index (PI), and pocket probing depth (PPD).

## 2. Materials and Methods

### 2.1. Patient Selection and Treatment

Between November 2022 and January 2024, a total of 128 patients underwent clinical examination for the treatment for gingival recession in a private clinic in Milan.

Inclusion criteria were as follows: single or multiple mandibular or maxillary RT1-type recessions, indication for class V restoration in combination with conservative and mucogingival surgical treatment, 18 years or older at the time of restorative treatment, patients’ informed consent, and willingness to allow data acquisition for the present study.

The exclusion criteria were as follows: the presence of ongoing orthodontic therapies, full-mouth plaque score (FMPS) ≥ 25%, full-mouth bleeding score (FMBS) ≥ 25%, pocket probing depth (PPD) ≥ 4 mm, as measured with a UNC-15 periodontal probe (University of North Carolina), the presence of a recession-associated frenulum that resulted in tissue ischemia when extended [22], substance or alcohol abuse, smoking [23], active oral infections, previous or recent radiation therapy in the oromaxillofacial area, recent chemotherapy, and pregnancy.

Patients completed an anamnestic questionnaire and underwent periodontal screening. Among the selected patients, a total of 50 teeth presented with buccal gingival recession at the buccal zenith (ZB), classified as recession type 1 (RT1). Seventeen of these teeth also presented with NCCLs characterized by the loss of enamel and dentin in the cervical region due to abrasion. The prognostic classification proposed by Pini Prato et al. [24] identified these lesions as class B+. Due to the clinical conditions, a two-phase treatment protocol was proposed: restorative reconstruction followed by re-evaluation at 4 months (±1).

The patient’s treatment process begins with an initial visit to assess general conditions and determine eligibility for participation. Suitable patients undergo a dedicated appointment where periodontal parameters are recorded and home hygiene motivation is evaluated.

A professional oral hygiene session follows, including additional motivational reinforcement and the collection of an alginate impression (T0). Motivational reinforcement was provided to ensure that patients adopted correct home oral hygiene techniques, aimed at preventing recurrence or worsening of recession and/or NCCLs [25]. One week later, a class V restoration is performed, providing another opportunity to reinforce patient motivation.

After four months, a follow-up visit is conducted. Periodontal parameters are reassessed, and a second alginate impression (T1) is taken to evaluate treatment progress and effectiveness over time.

All patients were initially treated using the BOVR technique alone. Surgical intervention was not part of the initial treatment protocol. It was considered only after the 4-month follow-up in cases where the restorative outcome was deemed unsatisfactory by both the clinician and the patient. The decision to proceed with surgery was not based on predefined clinical severity, but rather on the lack of sufficient tissue maturation following conservative therapy. This protocol allowed the conservative technique to be tested uniformly across all cases, minimizing allocation bias.

The study was conducted in accordance with the fundamental principles of the 1975 Helsinki Declaration on clinical analysis involving human subjects (revised in 2008) and was approved by the ethics committee of the Insubria University (#0118693) on 18 October 2023.

### 2.2. BOVR Technique Protocol

The BOVR technique involves composite cervical reconstruction to increase tooth thickness. The goal is not to replicate the original cervical anatomy lost due to the NCCL. Instead, the restoration must be thick enough to protect the gingival margin. The maximum extension reference is the MRC. The MRC was identified based on established clinical criteria [9]. Since the MRC position does not always match the original CEJ, this point is referred to as the dentin-composite junction (DTC). It represents the most apical point of the new CEJ, relative to the MRC. In traditional techniques, the restoration extends to the gingival margin, covering the exposed tooth surface [21]. The horizontal extension of the restoration was determined based on the gingival margin thickness at T0.

The first 0.5 mm coronal to the DTC was progressively extended to match the gingival thickness. This resulted in a cervical shape different from the original.

**Figure 1:**
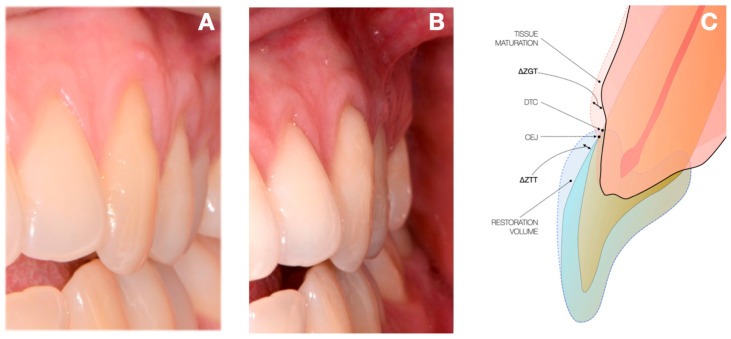
Illustration of the dental surface before (**A**) and after (**B**) the conservative therapy. Schematic drawing showing pre and post conservative therapy (**C**).

The horizontal thickness was designed to accomplish both anatomical and aesthetic requirements. It provides support for lip and cheek pressure [26] and restores a natural appearance. Achieving a smooth, continuous transition was essential; the restoration avoided any sharp angles and featured only polished, gradual curvature changes (Figure 1). One week before treatment, patients received an oral hygiene session. They also received motivation and guidance about proper oral hygiene. Patients were instructed to follow correct hygiene methods and eliminate bad habits such as incorrect brushing and flossing [27].

This protocol aimed to correct flat or concave emergence profiles at the buccal CEJ. Loss of cervical volume from erosion, abrasion, or abfraction was also addressed. The new restoration provided a modified cervical shape.

The dental surface was cleaned with pumice powder or glycine while under local anesthesia. The sclerotic dentin layer was removed with a rotating instrument. A retraction cord was inserted into the gingival sulcus (Ultrapak #000, Ultradent^®^, South Jordan, UT, USA). A rubber dam was then placed (Dental Dam, Nic Tone^®^, Bucarest, Romania).

Adhesive procedures included selective etching (OPTIBOND GEL ETCHANT, Kerr^®^, Brea, CA, USA) and a single-bottle adhesive system (OptiBond Solo Plus^®^, Kerr ^®^, Brea, CA, USA), following manufacturer instructions. Composite was applied incrementally, beginning with a dentin layer and followed by an enamel layer. Composite was intentionally over-contoured in the horizontal plane to enhance cervical convexity. Gingival margin thickness was used as a reference (Figure 1). This approach resulted in a thicker restoration than previously described [21], where the anatomical shape was simply restored. Composite resin selection prioritized long-term aesthetics [28].

Each composite layer was light-cured according to manufacturer guidelines. A second light-curing step was performed after applying an oxygen barrier (DeOx™, Ultradent^®^, South Jordan, UT, USA) to improve hardness [29].

Finishing was conducted with diamond burs and progressively finer diamond rubber polishers (COMPOSITE FINE, Shofu^®^, Ratingen, Germany). A polishing paste completed the process. Special care was taken to avoid gum damage from dam clamps or finishing tools.

To evaluate dental shape and tissue response, follow-up was conducted at 4 months (±1) (T1).

### 2.3. Thicknesses Data Collection

The initial conditions of the gingival tissues and dental shape at T0 were replicated by taking an impression with alginate, poured within 2 h using class IV dental stone, conserving the impression according to manufacturer instructions during this time. Only impressions with ideal accuracy of the region of interest were accepted. If bubbles or surface imperfections were observed in the alginate, the impression was repeated.

After 4 months (±1 month) (T1) new alginate impressions were then taken following the same procedures as at T0. The periodontal values were recorded as well at T1.

### 2.4. Periodontal Values Data Collection

Periodontal charting performed at T0 and T1 evaluated the following parameters: probing depth at the buccal zenith (PPDZB), maximum probing depth of the treated tooth (MAX PPD TOOTH), maximum probing depth across the entire dentition (MAX PPD MOUTH), plaque index (PI), and bleeding on probing (BOP) of the treated tooth.

The evaluation of the gingival biotype was carried out by classifying it into thin and festooned (type 1), intermediate (type 2), and flat and thick (type 3) as defined by of the periodontal probe transparency test [5,30].

The level of oral hygiene was assessed at T0 and T1 to evaluate the hygienic maintenance of the new tooth thickness by assessing the plaque index and the improvement or deterioration of tissue health after 4 months of conservative therapy. In cases where conservative treatment alone was insufficient to correct the extent of the recession, periodontal surgery was performed as a secondary corrective procedure. Eligibility for mucogingival surgery required a full-mouth plaque index (FMPI) and full-mouth bleeding on probing (FMBOP) below 20% [31].

The full-mouth plaque index was recorded as a percentage of the total surfaces (four aspects per tooth) with the presence of plaque shown by use of a plaque detector.

Bleeding on probing (BOP) was scored dichotomously under a pressure-sensitive manual probe calibrated at 0.3 N. The full-mouth bleeding index was recorded as a percentage of the total surfaces (four aspects per tooth) that showed the presence of BOP.

The PI and BoP of the studied tooth were recorded at T0, immediately before the first professional oral hygiene session, and at T1.

### 2.5. Digital Acquisitions, Orientation and Planes Settings

**Figure 2:**
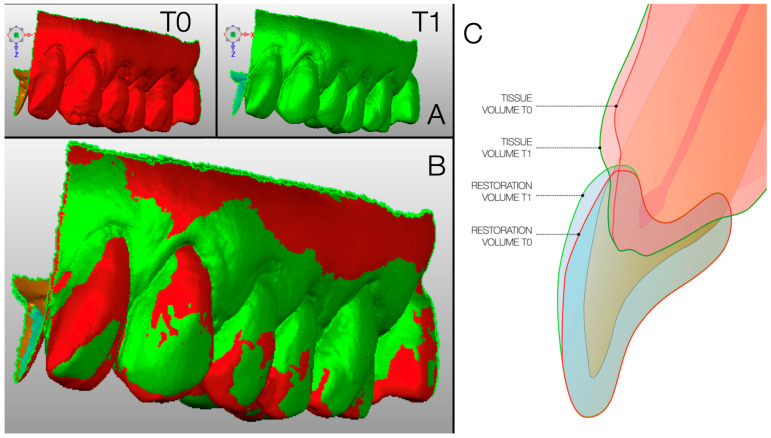
Comparison between T0 (before conservative therapy, on the left) and T1 (4 months after preserved therapy, on the right), both clinically and by 3D scanning of plaster models. (**A**) 3D surface models of dental casts at baseline (T0, in red) and after 4 months (T1, in green), shown separately for visual clarity. (**B**) Superimposition of T0 and T1 3D models to visualize morphological changes over time; red areas represent the baseline surface, while green areas represent the surface after 4 months. (**C**) Cross-sectional schematic representation of tooth and soft tissue volumes at T0 and T1, showing tissue and restoration volume changes over time.

The plaster models obtained at T0 and T1 were scanned with a laboratory scanner (Ceramill Map 400^®^, Amann Girbach^®^, Pforzheim, Germany). The resulting stereolithography (STL) files were collected for comparative analysis (Figure 2).

Three different software packages were used to analyze the digital data. Before performing the alignment procedures, the STL models were cleaned by removing irrelevant surface data to improve the quality of the alignment procedure using Geomagic^®^ Design X software version 2014.1.0 (3D Systems, Inc.^®^, Rock Hill, SC, USA). Alignment of T0 and T1 models was performed using this same software.

Superimposition was carried out in two distinct phases: first, a three-point point-based superimposition (PBS) was performed on the adjacent tooth surfaces, which allowed an initial rough three-dimensional surface alignment of the two models. Final refinement was achieved using the ‘best-fit’ surface alignment algorithm.

**Figure 3:**
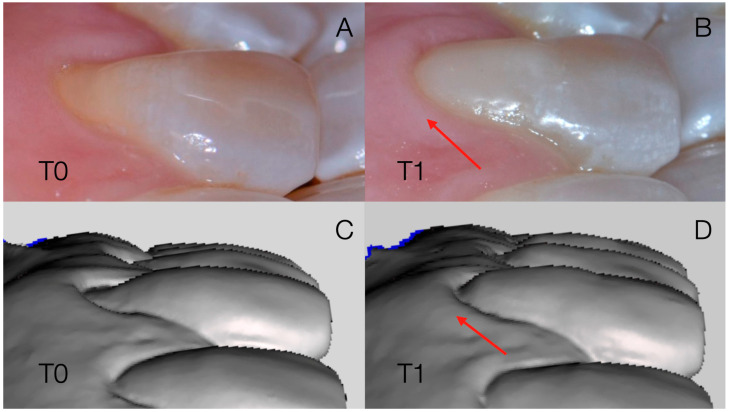
Sequence of model overlays at T0 and T1 (**A**,**C**) and subsequent matching (**B**,**D**) obtained using Geomagic3D software.

The digital measurements were performed by an operator who was blinded to the initial hypothesis of the study. Subsequently, an overlap threshold was set by requiring a minimum of 100 iterations in all cases to obtain the automatic overlap of the corresponding polygons. A robust iterative closest point (RICP) algorithm was used for this final recording and the distances between the overlap models were minimized using a point-to-plane method (Figure 3).

**Figure 4:**
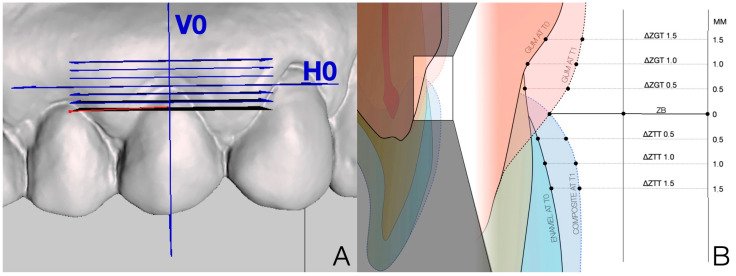
Position on the aligned models of the vertical cutting plane parallel to the long axis of the tooth (V0) and horizontal at the level of the buccal zenith (H0) (**A**). Schematic illustration of the different levels of elements after the alignment of the T0–T1 models (**B**).

Using Meshmixer^®^ software version 0.11.5.474 (Autodesk Ink.^®^, San Rafael, CA, USA), vertical and horizontal plane were created to align with the STL model generated from the superimposed T0 and T1 datasets. The vertical reference plane (V0) was positioned at the level of the buccal gingival zenith (ZB) and aligned with the tooth’s axial axis. The horizontal plane (H0) was perpendicular to V0 and ZB. The horizontal plane (H0) served as a reference for generating a series of parallel horizontal planes at 0.5 mm, 1 mm, and 1.5 mm from the H0 plane in both the apical and coronal directions (Figure 4).

### 2.6. Digital Analysis

**Figure 5:**
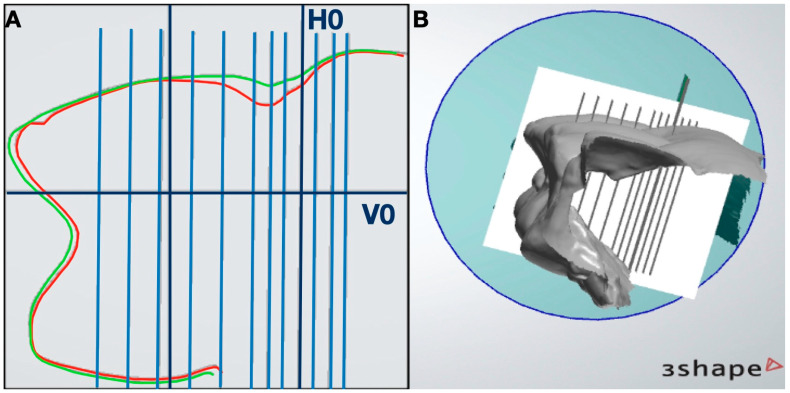
(**A**,**B**) show models at T0 (red line) and T1 (green line) superimposed using 3 Shape^®^ software. Model overlap shows increase in dental and gum thickness at T1.

Horizontal measurements were obtained by analyzing the intersections of the STL-based tooth and gingival profiles with the horizontal reference planes, as the points for the measurements of linear differences on the section planes between T0 and T1 were obtained using 3D Viewer software version 1.3.2.0 (3Shape^®^ Copenhagen, Denmark). These points represent the gingival and tooth profiles at the baseline and control times (Figure 5). By comparing intersections at T0 and T1, it was possible to quantify temporal changes in gingival maturation and composite contour.

Therefore, the soft tissue and the tooth were monitored in terms of horizontal changes relative to the section of plane. Changes in gingival thickness (ΔZGT) between T0 and T1 were measured with reference to the parallel horizontal planes. These planes were positioned both for the T0 and the T1 evaluation according to the ZB at T0, as described, in order to obtain a measurement that described the horizontal change at different distances from ZB. Soft tissue changes were described by ΔZGT according to the horizontal sectional planes at 0.5 mm, 1 mm, and 1.5 mm from ZB, obtaining 0.5-ΔZGT, 1-ΔZGT, and 1.5-ΔZGT, respectively. The delta relative to the change in tooth thickness, ΔZTT, was measured in the same way, on the coronal side to ZB at T1: respectively, 0.5-ΔZTT, 1-ΔZTT, and 1.5-ΔZTT. Both gingival and tooth measurements were considered positive in cases of volume gain and negative in cases of volume loss.

**Figure 6:**
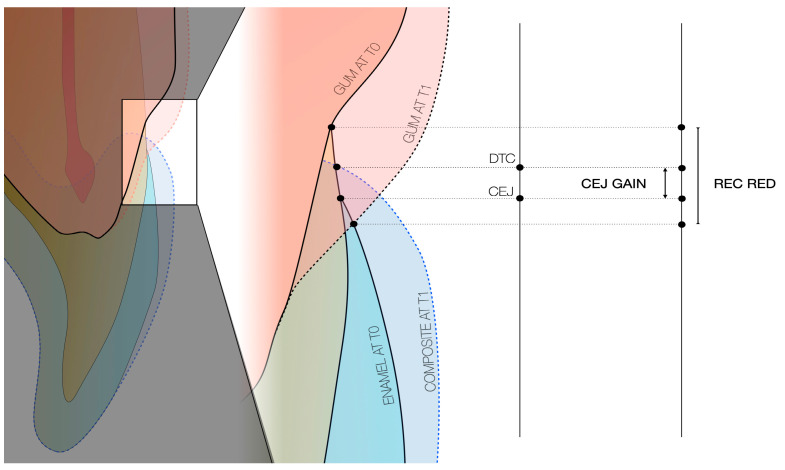
Tooth morphology after reconstruction.

Sagittal measurements: the digital analysis was also designed to evaluate the clinical change in crown height, interpreted as recession reduction (REC RED). It was obtained by calculating the distance from the incisal edge to the gingival margin (ZB at T0) along the V0 plane at T0 and T1. The REC RED between T0 and T1 was calculated to present positive values that indicated coronal migration of the free gingival margin, and negative values indicated apical migration (Figure 6).

### 2.7. Statistical Analysis

Statistical analysis was performed using SPSS 20 software (IBM© SPSS Statistics©, IBM Corp.© Armonk, NY, USA). Descriptive analysis was carried out firstly, analyzing mean values and standard deviations. Data were tested for normality of distribution (Shapiro–Wilk test) to choose the correct statistical analysis. The analysis aimed to assess the relationship between ΔZTT and ΔZGT at 0.5 mm, 1.0 mm, and 1.5 mm, as well as their association with REC RED. For the Shapiro–Wilk test, the null hypothesis was that the dataset was normally distributed. For *p* values < 0.05, the null hypothesis was rejected, as the sample was not normally distributed.

If one dataset exhibited a normal distribution and the other did not, a non-parametric test was chosen, as it is less sensitive to data distribution. The Wilcoxon signed-ranks test for paired samples was chosen to assess the relationship between the variables, and *p* values were calculated. The Rosenthal formula for calculating effect sizes r = Z/√N was then applied to the tests to assess the clinical significance of the results.

Rosenthal’s r was also converted to Cohen’s d to facilitate comparison with parametric effect size benchmarks. A Mann–Whitney U test was chosen for the periodontal values, as it suited the small sample size and the value distribution. Significant values were set with *p* ≤ 0.05 and a standard 95% confidence interval was applied. In previous similar studies, such as by Santamaria et al. [11], the sample size included 20 patients, divided into two groups; standard deviation values in their study ranged from 0.35 mm to 0.40 mm for changes in clinical attachment levels and gingival thickness. The intent was to detect a larger effect size or our study allowing detection of significant differences. Therefore, to achieve 80% power, aiming to detect significant changes in gingival tissue thickness and volume after the new method restoration in non-carious cervical lesions (NCCL), a sample of at least 47 cases was obtained. The sample size was designed to detect clinically relevant tissue alterations over the 4-month observation period with a confidence level of 95% and a beta error of 20%.

Of the 25 patients considered for treatment, 18 did not meet the inclusion criteria. Seven patients (mean age 35 ± 13 years, range 22–46) were selected by the periodontist as candidates for NCCL treatment. A total of 50 teeth with RT1 recession were then included in the study and underwent the conservative fifth class restoration. Of these, 29 (58%) dental elements were of type 1 biotype (thin and festooned) and 21 (42%) dental elements were of type 2 biotype (intermediate).

The distribution of the treated elements in the different sextants is shown in Table 1.

## 3. Results

### 3.1. Hygienic Maintenance Results

The purpose of this first analysis was to determine whether the increase in tooth thickness may be maintainable from a hygienic standpoint. Since the data were not normally distributed, a non-parametric test (the Wilcoxon signed-rank test) was applied.

A significant reduction (≤0.001) in PI was observed on the affected teeth. Table 2 shows a significant decrease (≤0.001) in the measurements of BOP, zenith PPD, and MAX PPD TOOTH between T0 and T1. The reductions in PI and BOP were significant at T1 compared to T0.

The results show that the increase in tooth volume at the buccal zenith, associated with correct hygiene instructions, did not result in an increase in PI, BOP, and PPD.

### 3.2. Gingival Thickness Results

ΔZTT and ΔZGT were compared at a standardized distance from ZB at T0 using digital overlays and reference planes to describe the volume behavior, mirroring the H0 plane.

Normality of data distribution was assessed using the Shapiro–Wilk test to inform the choice of appropriate statistical analysis. As the dataset was not normally distributed (Table 3), it was decided to use a non-parametric Wilcoxon test (Table 4).

Table 4 shows that the increase in tooth thickness corresponded to an increase in gingival margin thickness at the zenith.

Additionally, ΔZTT/ΔZGT at the three heights showed a ratio of 6.9:1 to 0.5 mm, 18.1 at 1 mm, and 19.1:1 at 1.5, respectively. The tooth thickness increase was more effective at the 0.5 mm level; nevertheless, an influence at the other measurements still exists. Consequently, a greater increase in tooth thickness was necessary to achieve clinically detectable gingival thickening.

Effect size analysis was used to determine whether the statistically significant findings (*p* ≤ 0.001) reported in Table 4 also held clinical relevance. A range of Cohen’s d values between −0.5 and −0.8 is generally considered to indicate a high level of clinical significance [32].

Effect sizes were calculated at all three measured distances from the ZB to validate the results (Table 5), and the observed correlations were considered clinically meaningful.

The effect size is reported as ‘d’ rather than ‘r’ because it was normalized using Cohen’s formula. This shows how the correlation is clinically significant.

### 3.3. Coronal Migration Results

ΔZTT and REC RED were compared at the same distance from the ZB at T0 to assess volumetric changes along the V0 plane.

The Shapiro–Wilk test was performed to assess the normality of the data distribution. Since the dataset was not normally distributed (Table 6), a non-parametric test—the Wilcoxon signed-rank test—was used for analysis (Table 7).

Analysis of Table 7 showed that the increase in tooth thickness corresponded to an increase in coronal migration of the gingival margin at the zenith.

Considering the ratio of ΔZTT/REC RED at the three measurement levels, a ratio of 3:1 to 0.5 mm was estimated. This suggests that a greater tooth thickness was required to achieve a greater coronal migration.

Influence between tooth modifications at a different distance from ZB on the recession reduction (*p* is calculated by the use of paired samples Wilcoxon signed-rank).

The effect size confirmed that the statistical significance (*p* ≤ 0.001) observed in Table 7 corresponded to clinically meaningful differences.

Effect sizes were calculated at the three distances from the ZB to validate the findings (Table 8), and the correlations were considered clinically significant.

That shows how the correlation is clinically significant.

## 4. Discussion

The increase in tooth thickness (ΔZTT) was significantly associated with an increase in gingival thickness (ΔZGT) at all three evaluated heights, as confirmed by the paired samples Wilcoxon signed-rank test (*p* ≤ 0.001). Therefore, the null hypothesis was rejected. The increase in tooth thickness (ΔZTT) corresponded to an increase in coronal migration (REC RED) with a significant correlation at all three evaluated heights as determined by the paired samples Wilcoxon signed-rank test (*p* ≤ 0.001). Therefore, the null hypothesis was accepted. The literature does not provide a clear answer on whether increased tooth thickness promotes plaque accumulation. Existing data on conservative therapy and hygienic maintenance remain inconclusive. Most studies focus on prosthetic rehabilitations, both on natural teeth and implants. In implants, cross-sectional studies suggest that a prosthetic interproximal contour above 30° increases the risk of peri-implantitis [33,34,35]. However, data specific to the buccal zenith area are lacking. Some studies contradict this correlation, reporting no relationship between wide contours and peri-implantitis, even at the buccal zenith [36,37]. A systematic review found no difference in hygienic maintenance between implant crowns with contours higher or lower than 30° [38]. For natural teeth, research suggests that crowns with an emergence profile up to 40° accumulate less plaque than non-prosthetically restored teeth [39]. However, there is no strong scientific evidence that increasing the transverse emergence angle at the CEJ improves hygiene. In this study, plaque control improved significantly after restoration (*p* < 0.001). These findings suggest that increasing tooth thickness may not lead to increased plaque accumulation and does not hinder cleaning [17,18,19,33,34,35]. The success of this hygiene maintenance may depend on the marginal angles of the prosthetic preparation, the smooth transitions, and the fact that the contour is buccal rather than interproximal. Increasing tooth thickness at the buccal zenith may help toothbrush bristles better clean the gingival margin. It could also protect against masticatory trauma. The new thickness shifts chewing pressure in a more vestibular direction, reducing impact at the cervical level [40]. Proper home oral hygiene contributes to these positive outcomes. Nevertheless, the present study does not preclude the possibility that the enhanced hygiene may be attributable to the novel tooth configuration or to enhanced patient oral hygiene practices. Indeed, untreated sites did not demonstrate analogous enhancements. No guidelines to date define an ideal emergence angle for maintaining hygiene on a reconstructed natural tooth. This study chose the evaluation interval without specific indications from the literature.

The position of the restoration margin and the gingival margin is important. It affects both the biological response and long-term stability. In the BOVR technique, the restoration is not placed at the original CEJ. The CEJ is often not visible due to abrasion. The margin is instead placed apically, at the level of the clinically determined MRC. This approach can have several advantages. It improves soft tissue support. It may allow passive gingival migration and protects the gingival margin. It avoids placing the margin too coronally, which could disturb tissue behavior. It also avoids placing it too apically, which may cause over-contouring and make hygiene more difficult [15,27]. There are some limitations. The MRC is a clinical estimate. It can vary with anatomical and biotype differences [41]. If the margin is placed inaccurately, tissue adaptation may be poor. In some cases, surgical correction may be needed. Some reports suggest waiting two weeks between restoration and periodontal surgery, without assessing gingival maturation during this period [14]. Others report gingival maturation 15 days after restoration [15]. Here, a longer interval was used to allow more time for tissue maturation. Given the null hypothesis that tooth shape influences gum response, this period effectively increased tissue thickness when combined with proper hygiene. This study measured gingival maturation on seven teeth, assessing changes in the clinical crown every 15 days through photographic analysis. The average gain was 0.267 mm, but this was not statistically significant (*p* = 0.88), possibly due to the small sample size [15]. At re-evaluation at 4 months, if the gingival maturation was not sufficient to obtain the root coverage deemed satisfactory by the clinician and the patient, then periodontal surgery was performed. The absence of this condition did not necessitate surgery. In this study, 50% of the treated elements did not require surgical correction.

In the present study, a REC RED gain of 0.12 mm (±0.2) was measured after four months using digital analysis. This suggests possible tissue maturation following restoration. No other studies report REC RED after cervical restorations, except in periodontal surgery cases. These results may reflect a biological behavior similar to what is described in the literature as creeping attachment. This term refers to the gradual coronal migration of soft tissue over exposed root surfaces. This phenomenon is mainly reported after mucogingival surgery, such as free gingival grafts. In these cases, an average soft tissue gain of about 0.66 mm has been observed after one month, especially in thick gingival biotypes [42]. A similar coronal shift has been seen in non-surgical cases. This was observed with the coronally dynamic technique [15]. The tissue changes seen in our study after four months may indicate a similar response. This could be linked to the new cervical morphology created by the restoration. More research is needed to understand the biological mechanisms. The primary goal of cervical reconstruction is restorative. However, its role in preparing gingival tissues for periodontal surgery is often overlooked. Unlike the “coronally dynamic” technique, the method used in this study does not require “gingitage” to induce a tissue response [15]. This makes the procedure less invasive and reduces the risk of damaging the sulcus. For this reason, no gingival bleeding is induced to stimulate tissue remodeling, which is a prerequisite in the BOPT technique [16]. Both BOVR and BOPT are based on the idea that tooth morphology can influence soft tissue behavior. BOPT is a prosthetic protocol. It is used for vertical tooth preparation and soft tissue conditioning around full crowns [16]. BOVR applies similar principles to conservative class V restorations. In this approach, the cervical emergence profile is over-contoured. The design depends on gingival thickness and does not reproduce the original anatomy. The intention is to promote passive gingival thickening and coronal migration over time. BOPT relies on provisional prostheses and gingival “gingitage” to guide tissue maturation [16]. In contrast, BOVR seeks to achieve similar biological effects using non-surgical restoration alone. BOVR can be viewed as a minimally invasive, restorative adaptation of BOPT’s biological concept. The technique encourages passive tissue maturation, promotes hygienic maintenance, and provides mechanical protection to the gingival margin. A conservatively reconstructed tooth with NCCL creates a convex, hard, and polished surface that supports the surgical flap margin [9]. This minimizes blood clot exposure to the oral environment, reducing the risk of destabilization by trauma and bacteria. More importantly, it prevents premature flap contraction, which could compromise surgical success. Supportive surfaces create space for blood clots between the flap and root, allowing proper maturation into connective tissue and improving long-term stability [9]. Tissue response resolved the recession, making mucogingival surgery unnecessary for both clinicians and patients. This suggests that, for smaller RT1 recessions, a conservative approach could be a viable alternative to surgery. In half of the cases, tissue repositioning occurred due to adaptation to the new tooth thickness, as proposed by the coronally dynamic technique [15].

Future research should explore whether increased tissue thickness could reduce the need for grafts, minimizing surgical trauma.

Studies show that after NCCL correction, using the coronally advanced flap (CAF) technique on keratinized tissue thicker than 0.8 mm produces nearly identical results, regardless of whether a connective tissue graft is used [13]. However, this study did not measure the emergence angle formed in the DTC, which remains a limitation.

### Study Limitations

One limitation is the tooth thickness-to-gingival thickness ratio. In this dataset, the ratio is 7:1 at the 0.5 mm gingival level, which has moderate clinical utility. Greater tooth thickness may be needed to achieve more noticeable clinical outcomes. The conservative approach adopted in this study may have been overly cautious. A more extensive application of composite material may enhance the gingival tissue response. Furthermore, the new emergence angle of the restoration was not measured in this study. Additional limitations include the small sample size (seven patients), the absence of a control group, and the uneven distribution of recessions across sextants. These factors restrict generalizability. Future research should also assess keratinized tissue height (KTH), a key parameter not considered here [43]. The mean recession size in this study was 1.66 mm (±0.79). Evaluating the technique’s effectiveness in larger recessions could improve its clinical relevance. Additionally, measurement accuracy could be refined. The 3D overlay technique and impression-based measurements have limitations. Future studies should incorporate intraoral scanners, which provide a more precise representation of oral structures [44,45]. Another important consideration is whether the four-month interval between procedures is optimal. A shorter period may suffice for tissue maturation, while a longer one could yield even better results. Alternatively, gingival thickening may regress over time, as observed in studies on periodontal surgery where outcomes were assessed at six and twelve months [46]. Future studies should investigate different time intervals.

## 5. Conclusions

Within the limitations of the study, the class V restorations performed using the BOVR technique may promote a positive soft tissue marginal adaptation. A 4-month maturation period, combined with CEJ anatomical reconstruction, increased tooth thickness at the zenith cervical region, and consistent hygiene routine may prevent the need for a surgical approach in present RT1 recessions.

In addition, the modifications to tooth shape did not compromise the patient’s ability to maintain oral hygiene, nor do they negatively affect periodontal health parameters. Further investigation is needed to evaluate additional variables and to include a larger sample size.

## Figures and Tables

**Table 1:** Subdivision teeth for sextant.

Subdivision Teeth for Sextant						
Sextant	I	II	III	IV	V	VI
n° teeth	9	11	11	6	4	9

I: upper right posterior sextant (teeth 14 to 17). II: central upper anterior sextant (teeth 13 to 23). III: left upper posterior sextant (teeth 24 to 27). IV: left lower posterior sextant (teeth 34 to 37). V: lower central anterior sextant (teeth 33 to 43). VI: right lower posterior sextant (teeth 44 to 47).

**Table 2:** Wilcoxon test comparing the mean variation for T0 and T1.

Comparison Between Periodontal Values at T0 and T1				
Value	T0	T1	DELTA	Sig
PI	0.72 ± 0.45	0.34 ± 0.48	−0.38 ± 0.53	≤0.001
BOP	0.7 ± 0.46	0.06 ± 0.24	−0.64 ± 0.48	≤0.001
ZENITH PPD	1.86 ± 0.35	1.42 ± 0.47	−0.44 ± 0.54	≤0.001
MAX PPD TOOTH	2.54 ± 0.5	2.38 ± 0.49	−0.16 ± 0.37	0.005
MAX PPD MOUTH	2.64 ± 0.48	2.64 ± 0.47	-	ns

Significance is set at *p* ≤ 0.05, ns means not significant.

**Table 3:** Shapiro–Wilk test at ΔZTT and ΔZGT.

Shapiro–Wilk Test ΔZTT-ΔZGT		
	ΔZTT	ΔZGT
0.5 mm	*p* = 0.02	*p* = 0.59
1 mm	*p* = 0.38	*p* = 0.00
1.5 mm	*p* = 0.44	*p* = 0.04

Shows a non-normal distribution.

**Table 4:** Influence between tooth and gum modifications.

Influence Between Tooth and Gum Modifications at 4 Months				
Level of Measurement	Tooth Changes (ΔZTT)	Gum Changes (ΔZGT)	Ratio ΔZTT/ΔZGT	Sig
0.5	0.381 ± 0.27	0.055 ± 0.11	6.9:1	≤0.001
1.0	0.525 ± 0.3	0.029 ± 0.14	18.1:1	≤0.001
1.5	0.573 ± 0.3	0.03 ± 0.11	19.1:1	≤0.001

*p* is calculated by the use of paired samples Wilcoxon signed rank.

**Table 5:** Effect size of the relationship between ΔZTT and ΔZGT at 0.5 mm, 1 mm, and 1.5 mm.

Effect Size ΔZTT-REC RED			
	0.5 mm	1 mm	1.5 mm
D	−0.5768	−0.6135	−0.612

**Table 6:** Shapiro–Wilk test at ΔZTT and REC RED.

Shapiro–Wilk Test ΔZTT-REC RED		
	ΔZTT	REC RED
0.5 mm	*p* = 0.022	*p* = 0.098
1 mm	*p* = 0.382	
1.5 mm	*p* = 0.442	

Shows a non-normal distribution.

**Table 7:** Influence between tooth modifications and coronal migration at 4 months.

Influence Between Tooth Modifications and Coronal Migration at 4 Months				
Level of Measurement	Tooth Changes (ΔZTT)	Coronal Migration (REC RED)	Ratio ΔZTT/REC RED	Sig
0.5	0.381 ± 0.27	0.124 ± 0.2	3:1	≤0.001
1.0	0.525 ± 0.3		4.2:1	≤0.001
1.5	0.573 ± 0.3		4.6:1	≤0.001

**Table 8:** Effect size of the relationship between ΔZTT and REC RED at 0.5 mm, 1 mm, and 1.5 mm.

Effect Size ΔZTT-REC RED			
	0.5 mm	1 mm	1.5 mm
D	−0.416	−0.5778	−0.582

## Data Availability

Available upon reasonable request.

## Abbreviations

BOVR	Biologically oriented V class restoration
CEJ	Cemento-enamel junction
PI	Plaque index score
BoP	Bleeding on probing score
ZB	Buccal zenith
H0	Horizontal plane perpendicular to V0
V0	Vertical plane aligned with the axial plane of the tooth
CEJ Gain	CEJ apicalization
REC RED	Clinical crown height variation between T0 and T1
ΔZTT	Delta related to the tooth thickness variation at different distances from the Buccal gingival zenith
ΔZGT	Delta related to the gingival thickness variation at different distances from the buccal gingival zenith
PPDZB	Probing depth at the buccal zenith
MAX PPD TOOTH	Maximum probing depth of the tooth
MAX PPD MOUTH	Maximum mouth probing depth
NCCL	Non-carious cervical lesions
MRC	Maximum root coverage
DTC	Dentine composite junction
PPD	Pocket probing depth
RICP	Robust iterative closest point
FMBS	Full-mouth bleeding score
FMPS	Full-mouth plaque (FMPS) score
